# Monoterpene Indole Alkaloids with Antimicrobial Activity Against *Helicobacter pylori*

**DOI:** 10.3390/ijms26167904

**Published:** 2025-08-15

**Authors:** Andreia T. Marques, Luís Tanoeiro, Angela Paterna, Maria Filomena Caeiro, David Cardoso, Silva Mulhovo, Joana S. Vital, Ana Carolina Pimentel, Maria-José U. Ferreira, Filipa F. Vale

**Affiliations:** 1 BioISI—Instituto de Biossistemas e Ciências Integrativas, Faculdade de Ciências, Universidade de Lisboa, Campo Grande, 1749-016 Lisboa, Portugal; andreia.f.marques@sapo.pt (A.T.M.); luistanoeiro@gmail.com (L.T.); joanamvital@gmail.com (J.S.V.); carolinapimentel2000@gmail.com (A.C.P.); 2 Research Institute for Medicines (iMed.ULisboa), Faculty of Pharmacy, Universidade de Lisboa, Av. Gama Pinto, 1649-003 Lisboa, Portugal; angela.paterna@gmail.com (A.P.); davidpcardoso@ff.ulisboa.pt (D.C.); mjuferreira@ff.ulisboa.pt (M.-J.U.F.); 3 cE3c—Centre for Ecology, Evolution and Environmental Changes, Faculdade de Ciências, Universidade de Lisboa, Campo Grande, 1749-016 Lisboa, Portugal; mfcaeiro@ciencias.ulisboa.pt; 4 Centro de Estudos Moçambicanos e de Etnociências (CEMEC), Faculdade de Ciências Naturais e Matemática, Universidade Pedagógica Campus de Lhanguene, Av. de Moçambique, Maputo 21402161, Mozambique; smulhovo@hotmail.com

**Keywords:** medicinal plants, *Helicobacter pylori*, cytotoxicity, antibiotic resistance, antibacterial activity, biofilm inhibition, mechanism of action, gastrointestinal diseases

## Abstract

*Helicobacter pylori* infection, a leading cause of gastric ulcers and gastric cancer, presents a major health challenge, exacerbated by rising antibiotic resistance. This study investigated the antibacterial potential of plant-derived compounds, isolated from different plant species, against *H. pylori*. Thus, a library of 153 natural compounds and derivatives, including monoterpene indole and bisindole alkaloids, obtained from the African medicinal plant *Tabernaemontana elegans* was screened in vitro for minimum inhibitory concentration (MIC) and minimum bactericidal concentration (MBC) against *H. pylori*. Active compounds (**1**–**7**) were tested for anti-biofilm activity and cytotoxicity on VERO cells to determine their half-maximal cytotoxic concentrations (CC_50_). Six monoterpene indole alkaloid azine derivatives (**1**–**6**) and vobasinyl-iboga type bisindole alkaloid (**7**) displayed antibacterial activity, with MICs between 10 and 20 µM. Compounds **2**, **3**, and **6** exhibited bactericidal activity, with MBCs of 20 µM. Notably, compounds **1** to **4** inhibited *H. pylori* biofilm formation at sub-inhibitory concentrations. Cytotoxicity assays revealed CC_50_ values above MICs, indicating a favorable safety profile for potential therapeutic use. This study highlights the potential of *T. elegans* monoterpene indole alkaloids as antibacterial agents and supports further exploration of plant-derived compounds as alternative treatments for *H. pylori*, offering a promising approach to address antibiotic resistance in gastrointestinal diseases.

## 1. Introduction

The Gram-negative bacterium *Helicobacter pylori* is recognized as the principal causative agent of severe gastric diseases, including peptic ulcers and gastric carcinoma. With its high prevalence, *H. pylori* is considered the most common bacterial pathogen in humans [1]. The most common treatment involves administering a proton pump inhibitor together with two antibiotics, usually clarithromycin plus either amoxicillin or metronidazole [2,3]. However, current eradication therapies face significant hurdles due to the rise in antibiotic resistance, which compromises treatment efficacy and highlights the urgent need for alternative solutions [4,5,6,7]. Consequently, discovering new treatments and safer inhibitory compounds has become a global health priority. Treatment efficacy may be further impaired by the presence of biofilms. Indeed, *H. pylori* strains have the capacity to form biofilms, adding a new layer of complexity to treatment and contributing to recurrent infections, reducing antibiotic efficacy and promoting survival [4]. *H. pylori* forms biofilms as stationary cell aggregates embedded in an extracellular matrix of proteins, DNA, and polysaccharides. Biofilm development typically begins with planktonic cells attaching to biotic or abiotic surfaces, resulting in a complex three-dimensional bacterial layer, while less common, non-surface-attached aggregate biofilms have also been observed [8].

Plants produce a wide variety of secondary metabolites, which not only support their survival but also possess biological properties such as antibacterial activity [9]. In fact, over two-thirds of the global population still relies on medicinal plants for primary healthcare [10]. Thus, plant-derived natural compounds have gathered increasing attention as promising sources of novel antimicrobial agents [11]. These compounds are valued for their diverse chemical structures and bioactivities, which can offer effective options against drug-resistant pathogens. Some medicinal plants have shown notable anti-*H. pylori* activity, with some active constituents, such as alkaloids, demonstrating strong efficacy comparable to standard antibiotics [12,13]. This potential makes plant-based research a critical avenue in the development of future anti-*H. pylori* therapies. Furthermore, natural compounds have been used for centuries in the treatment of gastrointestinal disorders like dyspepsia, gastritis, and peptic ulcer disease, all of which are caused by *H. pylori* [9,12]. Additionally, plant-derived natural compounds have been shown to inhibit biofilm production, a key factor in its antibiotic resistance and persistence [14]. The rapid extraction of plant species and the demand for alternatives to antibiotics have boosted the popularity of phytotherapy, making it a promising and emerging field in healthcare [9]. Alkaloids derived from medicinal plants hold significant promise as phytomedicines due to their diverse bioactivities and potential therapeutic effects; for instance, *Tabernaemontana* species are known to contain a rich variety of bioactive alkaloids with pharmacological potential [15,16,17].

*Tabernaemontana* species, traditionally used to treat several illnesses, are small to medium-sized trees widely distributed in tropical and subtropical regions of the world that can biosynthesize large quantities of monoterpene indole and bisindole alkaloids. Among indole alkaloids, terpenoid indole alkaloids are the largest group of secondary metabolites. They are an important class of *N*-heterocyclic compounds that have attracted attention due to their complex structure and bioactivities. Several compounds have been reported as antimicrobial agents inhibiting the growth of bacteria, fungi, and parasites [15]. Extracts and fractions rich in indole alkaloids were tested against bacterial strains, such as *Staphylococcus aureus*, *Escherichia coli*, *Klebsiella pneumoniae*, and *Pseudomonas aeruginosa*, revealing an antibacterial activity comparable to that of standard antibiotics such as ampicillin, cefoperazone, and imipenem [15]. Chen et al. (2022) also described indole alkaloids extracted from *Tabernaemontana* species showing antibacterial activity against *H. pylori* [18].

In this study, we focus on identifying natural plant-derived molecular inhibitors of *H. pylori* through the screening of a library of 153 compounds isolated from different plant species. This library encompasses in-house compounds, which have previously been reported, with various scaffolds, such as natural terpenoids (diterpenes and triterpenes), flavonoids, and alkaloids, and, mostly, corresponding semi-synthetic derivatives. The main goal was to explore their potential against this clinically significant pathogen. For compounds that demonstrated activity (**1**–**7**), additional assays, including anti-biofilm activity, cytotoxicity on cultured mammalian cells, and a preliminary mechanism of action assessment, were conducted to evaluate their safety profile and ensure their suitability for further development.

## 2. Results

### 2.1. Antibacterial Activity

**Figure 1:**
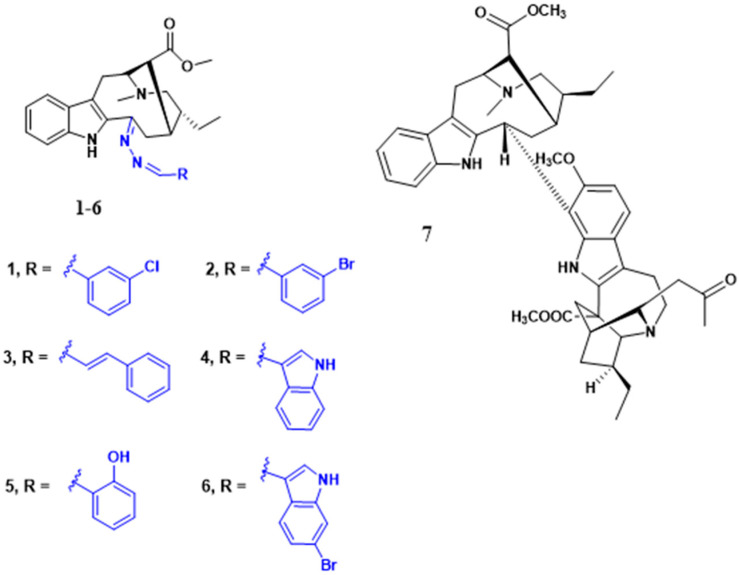
Structures of compounds **1**–**7**.

To identify bioactive compounds against *H. pylori*, we performed an initial screening of 153 compounds, with different scaffolds (Table 1), revealing seven active compounds (4.6%). These active compounds include six active monoterpene indole alkaloid derivatives, bearing an aromatic azine moiety (compounds **1**–**6**), and a vobasinyl-iboga type bisindole alkaloid (compound **7**) (Figure 1, Table 2). These indole alkaloids showed activity against *H. pylori* with minimum inhibitory concentration (MIC) and minimum bactericidal concentration (MBC) values between 10 and 20 µM (4.96–10.71 µg/mL). The solvent dimethyl sulfoxide (DMSO), at 1%, had no antibacterial effect.

All compounds exhibited MIC values of 20 µM or lower; compounds **1**–**3**, **5**, and **7** exhibited a MIC value of 20 µM (9.82, 10.71, 9.65, 9.45, and 15.25 µg/mL, respectively) whereas compounds **4** and **6** showed the most effective antimicrobial effect with a MIC value of 10 µM (4.96 and 5.75 µg/mL, respectively). Compounds **2**, **3**, and **6** had MBCs of 20 µM, indicating bactericidal activity. For compounds **1**, **4**, **5**, and **7**, MBCs could not be determined, suggesting a bacteriostatic effect at the tested concentrations (Table 2).

### 2.2. Anti-Biofilm Activity

**Figure 2:**
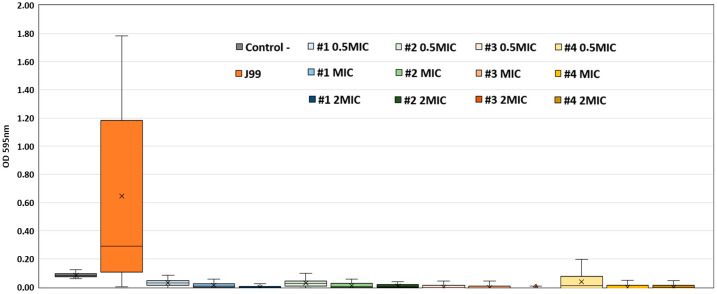
Anti-biofilm activity of compounds 1 to 4 in *H. pylori* J99. Each compound was tested at three concentrations: twice the MIC, the MIC, and half the MIC. *H. pylori* J99 biofilm formation without any compound is shown in orange. The control using culture medium only is shown in grey. The values for mean biofilm production and standard deviations were obtained from triplicate experiments (*n* = 3) for each compound concentration and controls. All comparisons between treated groups and the untreated control showed statistically significant differences (*p* < 0.001).

Biofilm formation was classified according to Fauzia et al. [25]. Briefly, a non-producer strain is classified when the OD 595 nm is lower than the OD 595 nm of the negative control; a weak producer when the OD 595 nm is between the OD 595 nm of the negative and 2× the OD 595 nm of the negative control and a strong producer is when the OD 595 nm is higher than 2× the OD 595 nm of the negative control. According to this criterion, *H. pylori* J99 was identified as a strong biofilm producer. We assessed the anti-biofilm activity of the tested compounds at twice the MIC, the MIC, and half the MIC, demonstrating that compounds **1** to **4** inhibited biofilm formation at all tested concentrations (Figure 2) in a likely dose-dependent manner. Following treatment, the OD 595 nm values decreased to levels corresponding to a non-producer phenotype, indicating strong anti-biofilm activity. ANOVA analysis revealed significant differences in biofilm formation among the tested groups (*p* < 0.001). Post-hoc Tukey’s test showed that compounds **1** to **4** significantly reduced biofilm formation compared to the untreated *H. pylori* J99 strain (*p* < 0.001). No significant differences were observed between compounds **5**, **6**, and the untreated strain. These findings confirm the inhibitory effect of compounds **1** to **4** on biofilm formation at sub-inhibitory concentrations.

### 2.3. Cytotoxicity Assay

**Figure 3:**
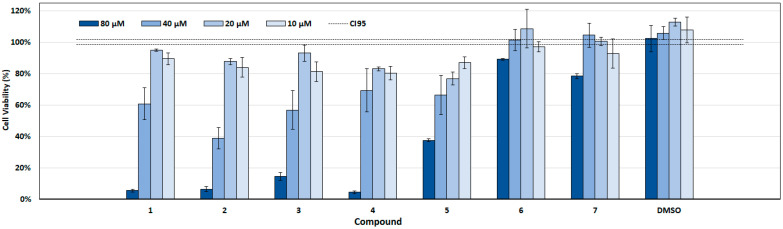
MTT cell viability assay on VERO cells after 48 h of exposure to serial concentrations (from 80 to 10 µM) of the various compounds. Also shown are the viabilities of cells treated with DMSO concentrations corresponding to those existing in the different concentrations of each compound. The mean viabilities (bars) and standard deviations from triplicate experiments (*n* =3) are depicted for each compound concentration and controls. The confidence interval at 95% (CI95) of the control is indicated by dashed lines. Statistical significance is not shown, for clarity; see main text for details.

Biosafety is a major key point to assess when exploring new potential therapeutic candidates. To address this, the cytotoxicity of each active compound was evaluated on VERO cells, for an exposure time of 48 h, with tested concentrations ranging from 10 to 80 μM (representing 2- to 10-fold the previously determined MICs, depending on the tested compound). Preliminary observation suggested that none of the seven active compounds tested led to noteworthy changes in cell adhesion or cell morphology, at MIC concentrations. Importantly, no statistically significant decrease in cell viability was observed in the DMSO control for any concentration (Figure 3), pointing to negligible biosafety concerns and excluding the potential negative effect of the solvent on the subsequent observations.

A summary plot of mammalian cell viability following exposure to all tested concentrations is shown in Figure 3. Exposure to compounds **1** to **5** significantly impacted cell viability at a concentration of 40 µM and above. Despite the impacts reported, none of the referred compounds displayed biologically relevant cytotoxicity for the bioactive concentrations of 10 and 20 µM. Notably, only a slight decrease in cell viability was observed at any of the concentrations tested for compounds **6** and **7**.

Eliciting their therapeutic potential, the exposition to 20 µM of each compound, which represents the MIC (for compounds **1**–**3**, **5**, and **7**) or 2-fold the MIC (compounds **4** and **6**), there was only a minimal reduction in cell viability across the compounds tested, indicating that the changes observed were not biologically relevant. In line, all compounds exhibited CC_50_ values for VERO cells higher than the MIC values determined for *H. pylori*, indicating that the compounds were not cytotoxic at the concentrations effective against the bacterium (Table 1). For compounds to which the dose–response curve could not be fitted to experimental data points due to a cytotoxicity under 50% for the highest concentration tested (80 µM), CC_50_ values are reported as >80 μM, which are clearly above the determined MIC values. The calculated Selectivity Index (SI) values (underestimated, considering that MIC was used, instead of the not determined but expectedly lower IC_50_ value) ranged between >1.7 and >8, indicating that the compounds demonstrate moderate to good selectivity toward bacterial cells relative to host cells. Statistically significant reductions in cell viability compared to both DMSO and untreated controls were observed for compounds **1** to **5** starting at 40 µM and continuing at 80 µM (*p* < 0.001), with compound **7** showing significant effects only at 80 µM. Compound **6** did not induce significant cytotoxicity at any tested concentration, indicating a favorable biosafety profile. At 20 µM and below, the cytotoxic effects were minimal or not statistically significant for most compounds, indicating a negligible biological impact at these concentrations. Overall, the cytotoxicity profiles plateaued between 40 and 80 µM, with no substantial increase in toxicity at 80 µM compared to 40 µM, suggesting that higher concentrations do not exacerbate cytotoxic effects.

Overall, the tested compounds showed no significant cytotoxicity at supra-inhibitory concentrations for *H. pylori* J99, under the assayed conditions, underlying their biological safety and potential as new therapeutic compounds against *H. pylori*, a major human pathogen.

### 2.4. Preliminary Assessment of Antibacterial Mechanism of Action

**Figure 4:**
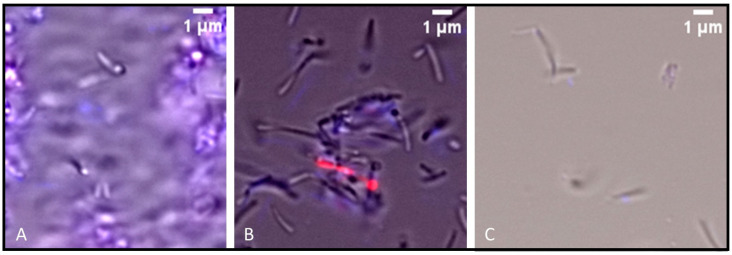
Hoechst/PI staining of *H. pylori* J99 after 72 h of exposure to (**A**) compound **5**, (**B**) compound **6**, and (**C**) untreated control. The bacterial samples in panels (**A**,**B**) were concentrated 5-fold compared to the control. Imaging was done on an Olympus BX60 fluorescence microscope (Olympus Corporation, Tokyo, Japan) with a 100×/0.3 objective coupled to a cooled Hamamatsu Orca R3 12-bit camera (Hamamatsu Photonics K.K., Hamamatsu City, Shizuoka, Japan). The fluorescence cube was Olympus U-MNIB, and spatially calibrated images were acquired using Micro-Manager 2.0 [26]. All images were processed for clarity and contrast (basic histogram adjustments) using ImageJ/Fiji version 1.54 g (developed by the National Institutes of Health, Bethesda, Maryland, USA) [27]. All imaging was performed at the Faculty of Sciences of the University of Lisbon’s Microscopy Facility, which is a node of the Portuguese Platform of BioImaging, reference PPBI-POCI-01-0145-FEDER-022122.

In the Hoechst/propidium iodide (PI) staining assay, untreated *H. pylori* cells exhibited high motility, with only a small fraction of residual cells staining positive for PI (<5%), indicating intact membrane integrity in the majority of the population. In contrast, treatment with the tested compounds resulted in a marked impairment of cell motility, with approximately 10% of cells staining positive for PI, suggesting limited membrane damage (Figure 4). Additionally, treatment with compounds **1** to **4** resulted in a pronounced reduction in cell number.

## 3. Discussion

The results obtained highlight the potential of plant-derived compounds, particularly indole alkaloids, as effective agents against *H. pylori*. Among the 153 compounds tested, 4.6% (7/153) of the compounds, namely six monoterpene indole alkaloid derivatives and one bisindole alkaloid, demonstrated antibacterial activity, with MIC values ranging from 10–20 µM. To the best of our knowledge, the antibacterial activity of these compounds has not been previously reported, making this study the first to evaluate their efficacy against *H. pylori*.

To prevent false positives, stringent endpoints should be considered, with the recommended half-maximal inhibitory concentration (IC_50_ values) being below 25 µM for pure compounds [28]. The IC_50_; is a quantitative measure that indicates the concentration of a substance required to inhibit a specific biological or biochemical function by 50%. Given that, by definition, MIC values are higher than IC_50_ values, the determined MIC endpoint aligns with this recommended cut-off, suggesting their potential efficacy as new antibacterial agents. The determined MBC values of the alkaloids were within the same dilution (for two compounds) or in the previous dilution (for one compound) of the respective MICs obtained for the bacterium, indicating that some antibacterial indole alkaloids from *T. elegans* (toad tree) are also bactericidal, thus possessing full antibacterial activity against the Gram-negative bacteria *H. pylori*. This suggests that specific structural features may be crucial for disrupting biofilm-related mechanisms. The observed anti-biofilm activity may be related to interference with *H. pylori* adhesion, biofilm matrix integrity, or quorum-sensing pathways, as reported for other alkaloids [29,30]. The cytotoxicity evaluation of these compounds against mammalian cells revealed CC_50_ values above the MIC values, indicating a favorable safety profile, i.e., showing antimicrobial activity without causing harm to mammalian cells at the tested concentrations. This is crucial for their potential application in therapeutic settings, where the goal is to eliminate pathogenic bacteria without damaging host tissues.

Interestingly, compounds **4** and **6**, having an extra indole moiety, exhibited the most potent antimicrobial effects, with MIC values as low as 10 µM, further supporting the idea that specific structural features of these alkaloids may contribute to their efficacy against *H. pylori*. Interestingly, compounds **4** and **6** contain indole systems, which are known to exert antibacterial effects through a combination of membrane disruption, oxidative stress induction, inhibition of DNA/protein synthesis, and enzyme targeting [31,32]. It is also noteworthy that compounds **6** and **7** showed no relevant biologic cytotoxicity at concentrations up to 80 µM, suggesting that they might have a wider therapeutic window. The SI values we observed, ranging from >1.7 to >8, are consistent with those reported in previous studies [33]. Furthermore, the observed SI values suggest that the compounds possess a reasonable degree of selectivity for bacterial cells over host cells, which supports their potential as viable candidates for further antimicrobial development, though optimization may be needed to enhance their therapeutic margin.

The preliminary insights into the potential mechanisms of action suggest that the antibacterial effects of the tested compounds are not primarily mediated by membrane disruption. The Hoechst/PI staining revealed that compounds **1** to **4** markedly reduced the number of viable *H. pylori* cells, with few cells visible under fluorescence microscopy. For all tested compounds the cells observed appeared highly immobile and largely unstained by PI, indicating that these compounds likely impair essential cellular functions or metabolic processes rather than compromising membrane integrity. Indeed, most cells remained PI-negative, though approximately 10% showed PI uptake, suggesting minor membrane perturbation. Further studies are required to elucidate the precise molecular targets involved.

Our findings align with previous studies highlighting the antimicrobial properties of plant-derived alkaloids, reinforcing their potential as alternatives to synthetic antibiotics. Namely, our compounds were from *T. elegans*, which is one of the *Tabernaemontana* species possessing antibacterial activity against Gram-positive bacteria and *Mycobacterium* species [34,35]. In fact, a variety of indole alkaloids have been isolated from different parts of this species, which exhibited biological activities, such as antimicrobial, antioxidant, anti-inflammatory, anticancer, and many others [15,23]. Previously, in an antimycobacterial evaluation of several African medicinal plants used in Mozambique traditional medicine to treat tuberculosis and related symptoms, the ethyl acetate extract of *T. elegans* exhibited significant activity against *Mycobacterium tuberculosis* H37RV (MIC 15.6 µg/mL) and H37Ra (MIC 31.2 µg/mL) and against *Mycobacterium bovis* BCG (MIC 15.6 µg/mL) [35].

## 4. Conclusions

In the present study, focused on indole alkaloids isolated from *T. elegans*, azine derivatives with a new aromatic ring in the indole alkaloid scaffold and a bisindole alkaloid showed notable antimicrobial activity. In particular, the most effective compounds, **4** and **6**, share an additional indole system, which seems to be responsible for their higher activity. These results also support the hypothesis that the indole alkaloids of *T. elegans* are responsible for the antimicrobial activity of this plant, and, in the future, these compounds can increase our drug arsenal to combat *H. pylori*. Further research, including studies and clinical trials, is needed to fully assess their therapeutic potential and safety for human use. Given the ongoing challenges of antibiotic resistance, the discovery of novel antimicrobial agents from natural sources remains a critical area of research in the search for new solutions to gastrointestinal diseases.

## 5. Materials and Methods

### 5.1. Natural Compounds Library

The library consists of in-house compounds previously reported [19,20,21,22,23,24], derived from various plant species and featuring diverse scaffolds, including natural terpenoids (diterpenes and triterpenes), flavonoids, alkaloids, and predominantly their semi-synthetic derivatives (Table 1). The alkaloid set was constituted mainly by monoterpene indole and bisindole alkaloids, isolated from the alkaloid fraction of the methanol extract of the roots of the African medicinal plant *T. elegans* (Apocynaceae), and derivatives, obtained by chemical modifications of major compounds [23,36]. Stock solutions of 1 or 5 mM were prepared in DMSO (Sigma-Aldrich, St. Louis, MO, USA).

### 5.2. In Vitro Valuation of Anti-H. pylori Activity

To evaluate the activity of the natural products library, in vitro assays were conducted to determine the MIC and MBC against the *H. pylori* reference strain J99. *H. pylori* was grown in *H. pylori*-selective medium (Biogerm, Porto, Portugal) or Brucella broth (Difco, Detroit, MI, USA) supplemented with 10% heat-inactivated fetal bovine serum (FBS, Gibco^TM^, Grand Island, NY, USA), at 37 °C, in a microaerophilic environment of 5% O_2_ and 10% CO_2_ (Anoxomat^®^, MART Microbiology BV, Lichtenvoorde, The Netherlands) for 48 h. The MIC value of each compound was determined in µM by the microdilution method using 2-fold serial dilutions ranging from 20 to 0.625 µM. An inoculum of 350 µL of a *H. pylori* suspension (OD 600 nm = 0.3) was added to each serial dilution for a final OD 600 nm of 0.15 in a final volume of 700 µL per well of a flat 48-well microplate (Sarstedt, Nümbrecht, Germany). The microplates were then incubated at 37 °C in microaerophilic conditions for 48 h with agitation of 140 rpm. After incubation, the optical density was read at 600 nm (BioTek Epoch2, Agilent, Winooski, VT, USA). All assays were performed in duplicate. DMSO at a concentration of 1% (*v*/*v*) was included as a negative control to assess the baseline effect of the solvent, in which bacterial growth was expected, while kanamycin (Sigma-Aldrich, St. Louis, MO, USA) at a concentration of 8 µg/mL was used as a positive control to provide a benchmark for effective antibacterial activity.

MIC values were defined as the lowest concentration of a compound that inhibited the growth of *H. pylori*. MBC values were determined by plating 5 µL aliquots of each dilution onto *H. pylori*-selective medium, followed by incubation as described above. MBC was defined as the lowest concentration of a compound that rendered no visible colony formation on the solid medium, indicating bactericidal activity.

### 5.3. Biofilm Formation Assay

*H. pylori* J99 was grown overnight in 10 mL of Brucella broth (Difco, Detroit, MI, USA) with 10% heat-inactivated fetal bovine serum (BB10) under agitation and microaerophilic conditions at 37 °C. Cultures were diluted to an OD 600 nm of 0.15 in BB10 medium, and 2 mL aliquots were prepared. Each compound (**1** to **7**) was added to the aliquots at three concentrations: twice the MIC, the MIC, and half the MIC. These were used to fill nine wells of sterile 96-well polystyrene microtiter plates. A similar procedure was followed for the positive control (bacteria only, without compounds). Each tested condition also included a negative control consisting of culture medium only. To assess the effect of DMSO, control wells containing equivalent DMSO concentrations (matching those in the MIC treatments) were included, with the same number of replicates. After static incubation at 37 °C for 5 days under microaerophilic conditions, the media was aspirated, and wells were washed twice with phosphate-buffered saline (PBS, 140 mM NaCl, 10 mM phosphate buffer, and 3 mM KCl, pH 7.4 at 25 °C). Crystal violet (0.1% wt/vol) was added for 2 min, then aspirated, and the wells were washed twice with PBS. Biofilms were visualized by adding 70% ethanol, and absorbance at 595 nm was measured. All experiments were performed in triplicate.

### 5.4. Cytotoxicity Assessment of Compounds

The cytotoxicity of the compounds showing antibacterial activity was evaluated in VERO cell cultures (FTVE, Vircell, S.L., Granada, Spain) using the standard MTT colorimetric assay [37,38]. Briefly, sub confluent cell cultures were exposed for 48 h, to 2-fold dilutions of each assayed compound, ranging from 80 to 10 µM, in 100 μL CO_2_-independent medium supplemented with 5% FBS and 50 mg/mL gentamicin (Gibco^TM^, Grand Island, NY, USA) (CO_2_-ind-FBS_5_). In each assay, cells exposed to equivalent concentrations of DMSO as solvent and untreated cells were kept as controls; untreated cells were considered the baseline for 100% viability.

After 48-h incubation at 37 °C, the culture medium was replaced by fresh CO_2_-ind-FBS_5_ medium supplemented with 0.5 mg/mL MTT (Thermo Fisher Scientific, Waltham, MA, USA). Upon a 3-h incubation at 37 °C, the medium was carefully discarded, and the purple formazan crystals formed were dissolved in DMSO (100 μL/well) during a 15-min incubation with agitation at room temperature, followed by absorbance reading at 570 nm, with reference wavelength at 630 nm (BioTek Epoch2, Agilent, Winooski, VT, USA). Data analysis and visualization were performed using Excel (v.2408) and RStudio (v. 2024.04.2). When possible, the half maximal cytotoxic concentration (CC_50_) values were determined for each compound by fitting a dose–response three- or four-parameter log-logistic function to the triplicate experimental points using RStudio (v.2024.04.2).

The selectivity index (SI) is the ratio of the cytotoxic concentration for 50% of the cells (CC_50_) to the inhibitory concentration for 50% of the bacteria (IC_50_). Considering that IC_50_ values were not calculated, but are expectably lower than the MIC value, the SI was estimated by dividing the CC_50_ by the MIC. Therefore, this value is necessarily lower than the SI because the MIC, rather than the bacterial half-maximal inhibitory concentration (IC_50_), was used in the calculation. This approach allows for an estimation of the therapeutic potential of the compound by providing an indication of its selectivity for targeting bacterial cells over host cells, even though the MIC is used instead of the bacterial IC_50_.

### 5.5. Membrane Integrity Assay in H. pylori

To assess the membrane integrity, each compound (at 1× MIC) was added to 500 µL of *H. pylori* J99 at an OD 600 nm of 0.15 in BB10 medium. The experiment includes wells only with bacteria (positive control) and with medium only (negative control). The cultures were grown for up to 72 h, at 37 °C, with agitation at 160 rpm, under microaerophilic conditions. Next, at 72 h, 100 µL of cells were subjected to Hoechst/PI double staining. Briefly, Hoechst (1 mg/mL stock, Sigma, St. Louis, MO, USA) and PI (2.5 mg/mL stock, Sigma, St. Louis, MO, USA) were each added at 1:100 (*v*/*v*) to liquid cultures of *H. pylori* in Brucella broth, followed by 15 min of incubation in the dark at room temperature. Cultures were then centrifuged, and the cell pellets resuspended in fresh Brucella broth. Samples were examined under an Olympus BX60 fluorescence microscope at 1000× magnification. In this assay, Hoechst dye stains the DNA of all cells, allowing visualization of the total bacterial population, while increased uptake of PI indicates compromised membrane integrity, as PI can only penetrate cells with damaged membranes. Thus, cells positive for PI are considered membrane-compromised, whereas Hoechst-positive/PI-negative cells are viable, with intact membranes.

### 5.6. Statistical Analysis

To compare biofilm formation between groups (negative control, *H. pylori* J99, and compound treatments), one-way analysis of variance (ANOVA) was performed, followed by Tukey’s post-hoc test for multiple comparisons. Data analysis was conducted using Python 3.13.5. Similarly, for the cytotoxicity assay in VERO cells, ANOVA and Tukey’s post-hoc test were also used to compare groups at each tested concentration (ranging from 80 to 10 µM), with DMSO-treated and untreated cells as reference groups.

## Figures and Tables

**Table 1:** Chemical scaffolds of terpenoids, flavonoids, and alkaloids screened against *H. pylori* through the screening.

Chemical Scaffold	Plant Species (Family)	Chemical Scaffold	Plant Species (Family)
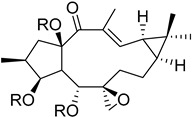 Macrocyclic lathyrane diterpenes and acylated derivatives	*Euphorbia boetica* Boiss. (Euphorbiaceae) [19]	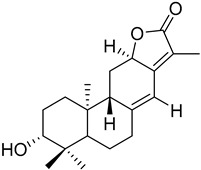 *ent*-abietane diterpenes	*Euphorbia piscatoria* Ait. (Euphorbiacae) [20]
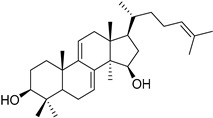 Tetracyclic triterpenes	*Cleistochlamys kirkii* (Benth) Oliv. (Annonaceae) [21]	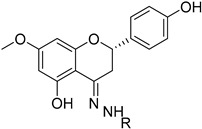 Flavonoid derivatives	*Euphorbia pedroi* (Euphorbiacae) [22]
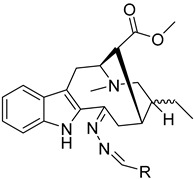 Monoterpene indole alkaloid azine derivatives	*Tabernaemontana elegans* (Apocynaceae) [23]	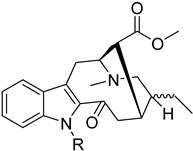 Monoterpene indole alkaloid alkylated derivatives	*Tabernaemontana elegans* (Apocynaceae) [23]
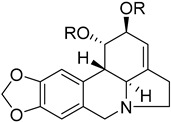 Amaryllidaceae-type alkaloids and derivatives	*Pancratium maritimum* L. (Amaryllidaceae) [24]	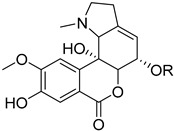 Amaryllidaceae-type alkaloids and derivatives	*Pancratium maritimum* L. (Amaryllidaceae) [24]

**Table 2:** Minimal inhibitory bactericidal concentrations of compounds against *H. pylori*, anti-biofilm activity, selectivity index (SI), and half-maximal cytotoxic concentration (CC_50_) values for the various compounds for VERO cells after a treatment of 48 h.

Compound	Name	MIC (MBC) (µM)	MIC (µg/mL) *	Anti-Biofilm Activity	CC_50_ (µM)	“SI” (CC_50_/MIC)
1	Dregamine(4′-chlorobenzylidene)hydrazone	20 (-)	9.82	+	44.1 ± 1.7	>2.2
2	Dregamine(4′-bromobenzylidene)hydrazone	20 (20)	10.71	+	34.8 ± 1.7	>1.7
3	Dregamine(phenylallylidene)hydrazone	20 (20)	9.65	+	44.1 ± 2.4	>2.2
4	Dregamine(indolylmethylene)hydrazone	10 (-)	4.96	+	47.0 ± 2.2	>4.7
5	Dregamine(2′-hydroxybenzylidene)hydrazone	20 (-)	9.45	-	55.8 ± 4.5	>2.8
6	Dregamine(6′bromo-3′indolylmethylene)hydrazone	10 (20)	5.75	-	>80 µM **	>8.0
7	Tabernaelegantinine A	20 (-)	15.25	-	>80 µM **	>4.0

* After conversion from µM to µg/mL; (-) MBC not determined, as bacterial growth was observed at all tested concentrations. MIC—minimal inhibitory concentration, MBC—minimal bactericidal concentration. ** For compounds to which the dose–response curve could not be fitted due to a value of cytotoxicity under 50% for the highest concentrations tested (80 µM), CC_50_ values are reported as >80 µM. “SI” instead of SI, because MIC values, and not IC_50_, were considered for the calculations.

## Data Availability

Data available on request. The data underlying this article will be shared on reasonable request to the corresponding author.
